# Survival of outborns with congenital diaphragmatic hernia: the role of protective ventilation, early presentation and transport distance: a retrospective cohort study

**DOI:** 10.1186/s12887-015-0473-x

**Published:** 2015-10-12

**Authors:** Katarina Bojanić, Ena Pritišanac, Tomislav Luetić, Jurica Vuković, Juraj Sprung, Toby N. Weingarten, William A. Carey, Darrell R. Schroeder, Ruža Grizelj

**Affiliations:** Division of Neonatology, Department of Obstetrics and Gynecology, University Hospital Merkur, Zagreb, Croatia; Department of Pediatrics, University of Zagreb, School of Medicine, University Hospital Centre Zagreb, Zagreb, Croatia; Department of Pediatric Surgery, University of Zagreb, School of Medicine, University Hospital Centre, Zagreb, Croatia; Department of Anesthesiology, Mayo Clinic, Rochester, MN 55902 USA; Division of Neonatal Medicine, Mayo Clinic, Rochester, MN USA; Division of Biomedical Statistics and Informatics, Mayo Clinic, Rochester, MN USA

**Keywords:** Acute lung injury, Comorbidity, Hernia, Diaphragmatic/epidemiology/mortality, Infant: newborn/outborn status, Mechanical ventilation: pressure controlled/volume controlled, Risk assessment, Severity of illness index: probability of survival score

## Abstract

**Background:**

Congenital diaphragmatic hernia (CDH) is a congenital malformation associated with life-threatening pulmonary dysfunction and high neonatal mortality. Outcomes are improved with protective ventilation, less severe pulmonary pathology, and the proximity of the treating center to the site of delivery. The major CDH treatment center in Croatia lacks a maternity ward, thus all CDH patients are transferred from local Zagreb hospitals or remote areas (outborns). In 2000 this center adopted protective ventilation for CDH management. In the present study we assess the roles of protective ventilation, transport distance, and severity of pulmonary pathology on survival of neonates with CDH.

**Methods:**

The study was divided into Epoch I, (1990–1999, traditional ventilation to achieve normocapnia), and Epoch II, (2000–2014, protective ventilation with permissive hypercapnia). Patients were categorized by transfer distance (local hospital or remote locations) and by acuity of respiratory distress after delivery (*early presentation*-occurring at birth, or *late presentation*, ≥6 h after delivery). Survival between epochs, types of transfers, and acuity of presentation were assessed. An additional analysis was assessed for the potential association between survival and end-capillary blood CO_2_ (P_c_CO_2_), an indirect measure of pulmonary pathology.

**Results:**

There were 83 neonates, 26 in Epoch I, and 57 in Epoch II. In Epoch I 11 patients (42 %) survived, and in Epoch II 38 (67 %) (*P* = 0.039). Survival with early presentation (*N* = 63) was 48 % and with late presentation 95 % (*P* <0.001). Among early presentation, survival was higher in Epoch II vs. Epoch I (57 % vs. 26 %, *P* = 0.031). From multiple logistic regression analysis restricted to neonates with early presentation and adjusting for severity of disease, survival was improved in Epoch II (OR 4.8, 95%CI 1.3–18.0, *P* = 0.019). Survival was unrelated to distance of transfer but improved with lower partial pressure of P_c_CO_2_ on admission (OR 1.16, 95%CI 1.01–1.33 *per* 5 mmHg decrease, *P* = 0.031).

**Conclusions:**

The introduction of protective ventilation was associated with improved survival in neonates with early presentation. Survival did not differ between local and remote transfers, but primarily depended on severity of pulmonary pathology as inferred from admission capillary PcCO_2_.

## Background

Congenital diaphragmatic hernia (CDH) is a congenital malformation associated with life-threatening pulmonary dysfunction and high neonatal mortality. In neonates with CDH the presence of respiratory distress after delivery indicates severe pulmonary involvement that requires medical management which includes mechanical ventilatory support prior to undergoing surgical correction [1].

The University Hospital Center (UHC) in Zagreb Croatia is the national treatment center for neonates with CDH. Before 2000, neonates with CDH cared for at UHC underwent intermittent mandatory ventilation (IMV) to achieve hyperventilation, a strategy based on the premise that respiratory alkalosis may help control degree of pulmonary hypertension [1–3]. However, ventilation of hypoplastic lungs with high tidal volumes may induce volutrauma associated with intra-alveolar hemorrhage and interstitial pulmonary edema [4–6]. It was subsequently determined that protective ventilation which uses the minimal pressure and volume settings to achieve acceptable oxygenation while allowing for hypercapnia can reduce lung injury [7–11]. Therefore, in the year 2000 UHC adopted protective ventilation for neonates with CDH.

The UHC lacks a maternity ward, thus all neonates with CDH are either transferred from local Zagreb hospitals or remote areas in Croatia. This practice is concerning because remote transfer of CDH neonates may increase mortality [12]. The primary aim of this study was to estimate the effects of the introduction of protective ventilation on survival. A secondary aim was to examine whether remote transfer, compared to local transfer, impacted survival.

## Methods

This study was approved by the Institutional Ethics Committee of the University Hospital Centre (UHC), Zagreb, Croatia. Due to retrospective design of this study the written consent was waived by the UHC Institutional Ethics Committee.

### Study design

A retrospective cohort study of neonates with CDH born between January 1, 1990 and December 31, 2014 who were treated at a single institution. The primary aim was to assess whether the survival of neonates with CDH improved after the year 2000 with the introduction of protective ventilatory strategy. Since neonates with early presentation are expected to have lower survival, the effects of protective ventilation both overall and in the subset of neonates with early presentation was assessed. The secondary aim was to explore the association between the type of transfer (local vs. remote) and survival. Because the time period covered by conventional ventilatory strategies was characterized by a national conflict (Croatian War of Independence 1991–1995) and travelling was hampered, this association was explored following the introduction of protective ventilation.

### Study setting

The neonatal intensive care unit (NICU) of University Hospital Center, Zagreb Croatia. The UHC is the national treatment center for neonates with CDH. UHC lacks a maternity ward; therefore all subjects in this study are outborns.

### Definitions

*Epoch I* is the period between January 1, 1990 and December 31, 1999, during which time hyperventilation with non-synchronized ventilation was used. *Epoch II* is the period between January 1, 2000, and December 31, 2014, during which time a permissive hypercapnia using protective ventilation was used. *Outborn status* refers to infants born in another hospital requiring transport to higher level of care. Patients were defined as “local transfers” if they came from local Zagreb hospitals or “remote transfers” from the rest of the country. *Early presentation* is when respiratory distress is evident immediately after delivery requiring endotracheal intubation and mechanical ventilation. *Late presentation*, on the other hand, is when the onset of breathing difficulties is delayed >6 h after delivery.

### Management strategies

#### Epoch I

Neonates were sedated, paralyzed, and ventilated with intermittent mandatory ventilation (IMV) to achieve respiratory alkalosis and postductal oxyhemoglobin saturation above 90 % to ameliorate pulmonary hypertension. This strategy often required higher peak inspiratory pressures (PIP), respiratory rates and oxygen concentrations. In those with available records of PIP, the values were between 30 and 40 cmH_2_O.(new line and header) Epoch II (new line) Ventilation was protocolized, and all neonates received protective ventilation aimed to minimize volutrauma with the use of minimal pressure and volume settings and inspired oxygen concentration to achieve acceptable preductal oxygenation saturations (≥85 %) while permitting hypercapnia (≤65 mmHg). Only two modes of ventilation were used during this time period: assist-control plus volume limit mode (A/C + VL) and pressure support ventilation with volume guarantee mode (PSV + VG). Both modes fully supported synchronized ventilation aided by controlled “demand flow” circuitry which synchronizes inspiratory gas delivery close to the breathing pattern of the neonate. Ventilatory settings were set *per* protocol. In the A/C + VL mode the tidal volume limit was 6 mL/kg, PEEP of 2–3 cm H_2_O, PIP ≤ 25 cmH_2_O, and the backup respiratory rate 40 *per* min. If respiratory acidosis (obtained from preductal capillary blood) was present (pH <7.25, PcCO_2_ >65 mmHg), ventilatory settings were changed by increasing PIP by 2 cmH_2_O (until maximum PIP of 25 cmH_2_O was achieved). In patients ventilated with PSV + VG mode the mean VG used was 4.0 mL/kg (range 2.6–5.5 mL/kg), PEEP 3.8 (range 2.5–5) cm H_2_O, PIP ≤ 25 cmH_2_O, and backup respiratory rate 40/min. If severe respiratory acidosis was present, VG was increased to a maximum 5.5 mL/kg exceeding the PIP limit if needed. With this strategy sedation and muscle paralysis were infrequently used and only in newborns with patient-ventilator asynchrony. High frequency oscillation ventilation (HFOV) was a rescue treatment for neonates who continued to have hypoxia and hypercarbia (PcCO_2_ >65 mmHg) despite optimization of either ventilatory mode. During Epoch II inhaled nitric oxide (iNO) became available and was used for neonates with ductal shunting (difference between preductal and postductal oxygen saturation >5 %), refractory preductal hypoxemia (PcO_2_ <60 mmHg with FiO_2_ >80 %), and for elevated right ventricular pressures. Surgical repair was typically done following initial optimization of respiratory parameters.

### Data collection

Patient variables that were abstracted included demographic information (date of birth, sex, place of birth [local vs. remote transfers]); birth information (gestational age, birth weight, Apgar scores); CDH information (prenatal diagnosis, acuity of presentation, pulmonary hypertension, type of CDH, presence of peritoneal sac and diaphragmatic aplasia), and physiologic variables obtained early during hospitalization (admission preductal capillary blood gases, lowest body temperature and lowest mean blood pressure within 12 h of admission). Probability of survival (POS) was assessed from the equation proposed by the Congenital Diaphragmatic Hernia Study Group [13], to categorize neonates into 3 POS score groups based on birth weight and 5-min Apgar score: low (0 %–33 %), moderate (34 %–66 %), and high (67 %–100 %) predicted survival groups. Variables regarding CDH management abstracted included mechanical ventilation mode; occurrence of preoperative pneumothorax; use of iNO, surfactant, and/or vasoactive support; type of surgical repair (primary vs. non-primary with patch); and time between delivery and surgery. Survival to hospital discharge was noted.

### Statistical analysis

Data are presented using mean ± SD or median [25^th^, 75^th^ percentile] for continuous variables, and frequency percentages for categorical variables. Characteristics were compared between groups using the 2- sample *t*-test, rank sum test, Chi square test, or Fisher’s exact test. Logistic regression was used to assess whether hospital survival was associated with epoch after adjusting for POS score. In order to assess for trends in survival over time, before and after the introduction of the protective ventilation, logistic regression analyses was performed for each time period with hospital survival as the dependent variable and calendar year as the continuous explanatory variable. To explore the association between local vs. remote transfer on survival we focused on neonates with early presentation of symptoms during Epoch II. Survival was compared using the Chi square test. In all cases 2-tailed P values <0.05 were considered statistically significant. Data were analyzed using SAS version 9.3 (SAS Institute Inc, Cary, NC).

## Results

**Fig. 1:**
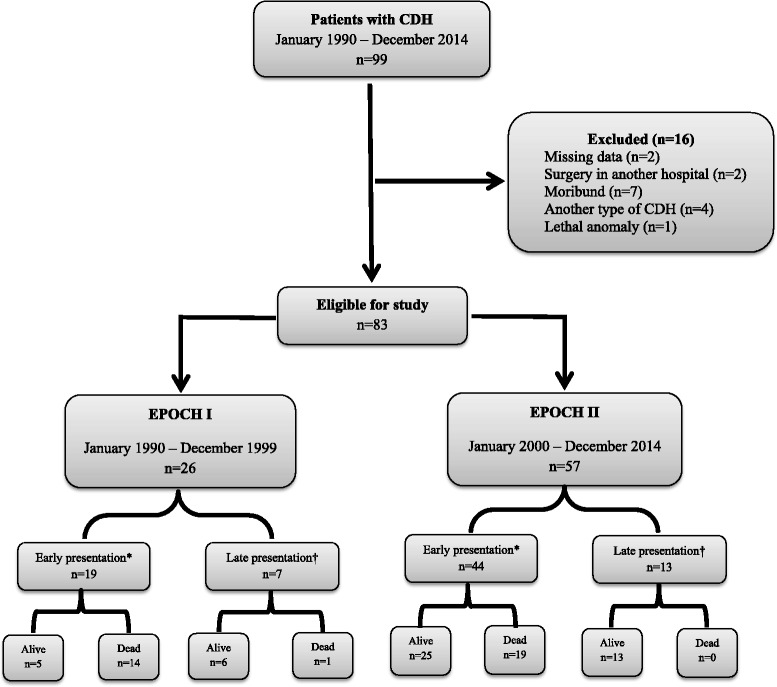
Patients with congenital diaphragmatic hernia. Exclusions: other types of congenital diaphragmal hernia (*n* = 4): Morgagni hernia, paraesophageal hernia, central hernia, severe diaphragmatic eventration. Lethal anomaly (*n* = 1) Edwards syndrome (trisomy 18) *Early presentation is defined as respiratory distress immediately after birth requiring endotracheal intubation; ^†^Late presentation is defined as respiratory distress either absent or present >6 h after delivery

Between January 1, 1990, and December 31, 2014, there were 83 neonates who received formal intensive care treatment, 26 were treated in Epoch I, and 57 in Epoch II. Sixteen additional patients were excluded for various reasons (see Fig. 1). All neonatal transfers to our medical center were accomplished by ground ambulance. Ventilation during transfer was accomplished via hand-held self-inflating bags for all neonates in Epoch I, and for the majority of neonates in Epoch II. In Epoch II, 9 neonates, 1 local (survived) and 8 remote (3 died) transfers, received ventilation through a pressure controlled ventilator integrated in transport incubator.

Table 1 summarizes demographic and disease characteristics of neonates with CDH. Table 2 summarizes admission capillary blood gases and vital signs within 12 h of admission. Table 3 summarizes medical and surgical interventions. During Epoch I all neonates received IMV, while in Epoch II protective ventilation was utilized, including 8 patients who received rescue HFOV. Surfactant and vasoactive support increased and iNO became available during Epoch II.

**Table 1:** Demographic and disease characteristics in children with congenital diaphragmatic hernia (CDH)

Characteristic	Overall	Epoch I	Epoch II	*P*-value
	(*N* = 83)	(*N* = 26)	(*N* = 57)	
Prenatal diagnosis of CDH^a^	21 (25)	1 (4)	20 (35)	<0.001
Sex				0.888
Male	52 (63)	16 (62)	36 (63)	
Female	31 (37)	10 (38)	21 (37)	
Gestational age, weeks	38.6 ± 2.5	39.0 ± 2.0	38.4 ± 2.7	0.318
Birth weight, kg	3.1 ± 0.6	3.1 ± 0.6	3.1 ± 0.6	0.909
Small for gestational age	4 (5)	1 (4)	3 (5)	1.00
Apgar 1 minute	6.4 ± 2.8	7.1 ± 2.6	6.1 ± 2.9	0.129
Apgar 5 minute	6.5 ± 2.7	6.5 ± 2.8	6.5 ± 2.7	0.920
Early presentation^b^	63 (76)	19 (73)	44 (77)	0.684
Local transfers	47 (57)	10 (38)	37 (65) ^b^	0.024
CDH type				0.661
Left	70 (84)	21 (81)	49 (86)	
Right	12 (15)	5 (19)	7 (12)	
Bilateral	1 (1)	0 (0)	1 (2)	
Probability of survival score (%)	0.63 ± 0.26	0.63 ± 0.24	0.63 ± 0.26	0.988
High (67–100)	43 (52)	14 (54)	29 (51)	
Moderate (34–66)	29 (35)	9 (35)	20 (35)	
Low (0–33)	11 (13)	3 (11)	8 (14)	
Pneumothorax (preoperative)	13 (16)	4 (15)	9 (16)	0.962
Pulmonary hypertension				<0.001
Not assessed	20 (24)	20 (77)	0 (0)	
Present	33 (40)	1 (4)	32 (56)	
Absent	30 (36)	5 (19)	25 (44)	
Diaphragmal aplasia	7 (8)	3 (11)	4 (7)	0.672
Peritoneal sac present	8 (10)	3 (11)	5 (9)	0.701

Data are N (%) or mean ± SD

^a^All prenatally diagnosed CDH from remote areas were transferred to Zagreb, 1 in Epoch I, and 15 in Epoch II

^b^Respiratory distress at birth

**Table 2:** Admission capillary blood gases, lowest mean blood pressure and lowest temperature over the first 12 h after admission

Characteristic	Epoch I		Epoch II		*P*-Value
	N	Mean ± SD	N	Mean ± SD	
PcO_2_, mmHg	21	55.8 ± 25.0	57	52.3 ± 19.5	0.521
PcCO_2_, mmHg	20	57.3 ± 19.3	57	70.4 ± 30.9	0.080
pH	22	7.17 ± 0.19	54	7.16 ± 0.22	0.831
Base deficit, mEq/L	20	−5.82 ± 6.39	54	−5.95 ± 7.43	0.946
Lowest temperature, °C	19	36.3 ± 0.4	51	36.1 ± 0.6	0.228
Lowest mean blood pressure, mmHg	19	42.0 ± 7.6	56	38.4 ± 8.1	0.094

Abbreviation: *PcCO*_*2*_ partial pressure of carbon dioxide in the end-capillary blood

**Table 3:** Interventions and type of surgical repair in neonates with congenital diaphragmatic hernia

Characteristic	Epoch I	Epoch II	*P*-value
	(*N* = 26)	(*N* = 57)	
Primary mechanical ventilation, n (%)			<0.001
Intermittent mandatory ventilation	26 (100)	0 (0)	
Assist-control + volume limit mode	0 (0)	26 (45)	
Pressure support + volume guarantee mode	0 (0)	31 (55)	
High frequency oscillatory ventilation^a ^	0 (0)	8 (15)	----
Inhaled nitric oxide	0 (0)	31 (54)	<0.001
Surfactant administration	1 (4)	16 (28)	<0.001
Vasoactive support	13 (50)	55 (96)	<0.001
Died before surgery	7 (27)	12 (21)	0.815
Time between delivery and surgery, hours^b^	24.5 [24.7, 28.2]	29 [23.0, 29.0]	0.550
Type of surgical repair	19 (73)	45 (79)	0.815
Primary closure	18	39	
Patch repair	1	5	
Muscle flap repair	0	1	

All values are N (%) or median [25^th^, 75^th^ percentile]

^a^Used only as a rescue technique

^b^Although current practice shifted from emergent repair of CDH to a policy of preoperative medical stabilization using a variety of intensive care management strategies, a recent Cochrane analysis showed that there was no clear evidence which favors delayed versus immediate (within 24 h of birth) surgical intervention [29]

### Overall cohort survival

In Epoch I 11 of 26 patients (42 %) survived to discharge, compared to 38 of 57 (67 %) in Epoch II (OR = 2.7, 95 % CI 1.1 to 7.1, *P* = 0.039 for survival during Epoch II vs. Epoch I). The percentage of patients who died after being admitted without surgery was similar between Epochs I and II, 27 % vs. 21 %, respectively (*P* = 0.815). Among those who were discharged, length of stay did not differ significantly between Epoch I vs. Epoch II (37 [19, 55] vs. 34 [23, 67] days; rank sum test *P* = 0.615). Calculated POS score negatively correlated with admission end-capillary partial pressure of carbon dioxide, P_c_CO_2_ (r = −0.35, *P* = 0.008). Survival was similar for patients who were local vs. remote transfers, (53 % vs. 67 %, *P* = 0.216). No temporal trends in survival were observed over calendar time during Epoch I (*P* = 0.490) or Epoch II (*P* = 0.373). From an analysis restricted to Epoch II, there was no difference in survival between neonates who were prenatally diagnosed with CDH compared to those without prenatal diagnosis (*P* = 0.174).

### Early vs. late presentation survival

**Fig. 2:**
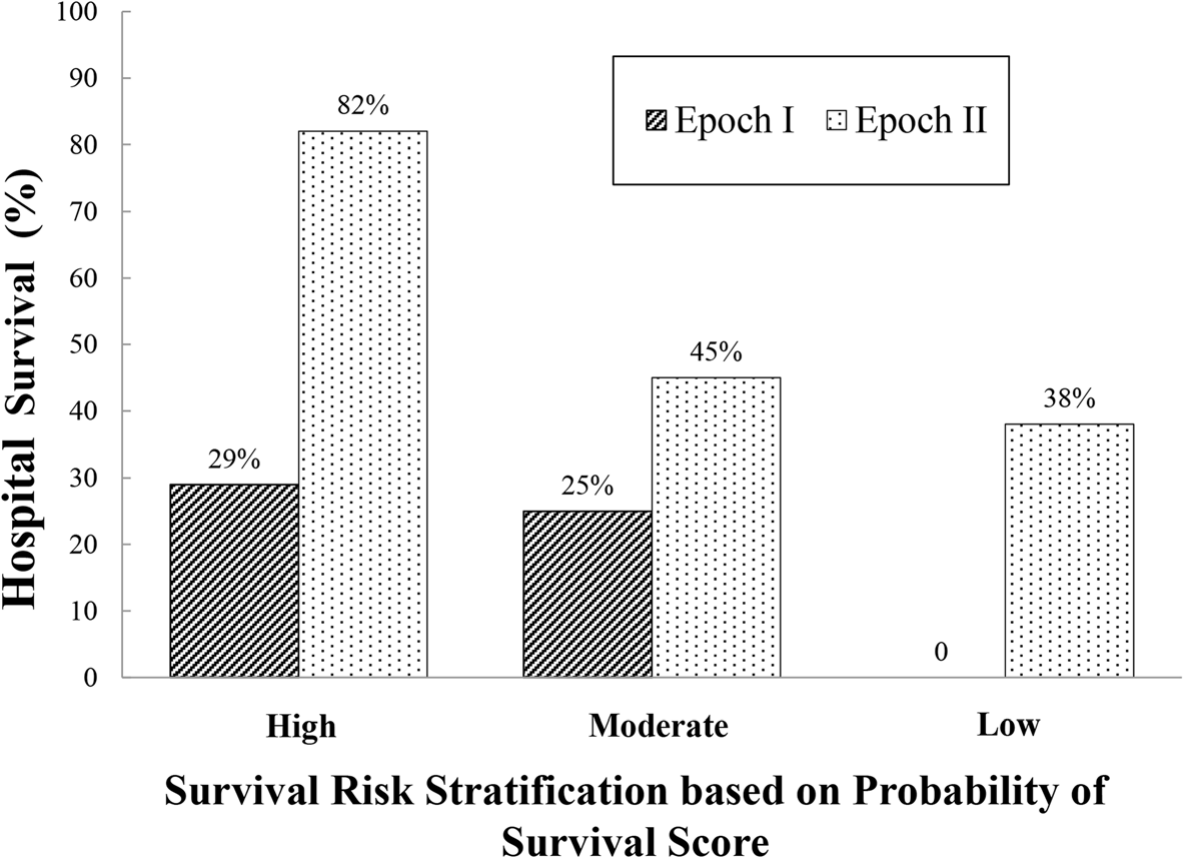
Hospital survival in neonates with early presentation of respiratory distress for Epoch I and II according to expected survival (low, moderate and high) based on calculated probability of survival score (see Methods). Within each risk stratification group there was a large increase in survival in Epoch II

Sixty-three neonates had early presentation and their survival was worse compared to those with late presentation (48 % vs. 95 % survival, *P* <0.001). Among early presentation neonates, survival was higher in Epoch II vs. Epoch I (25 of 44, 57 % vs. 5 of 19, 26 %, OR 3.7 95 % CI 1.1–12.0, *P* = 0.031). From multiple logistic regression analysis restricted to early presentation neonates and adjusting for POS score, survival improved in Epoch II compared to Epoch I (OR 4.8, 95 % CI 1.3–18.0, *P* = 0.019). In an analysis restricted to neonates with early presentation, no temporal trends were observed over calendar time during Epoch I (*P* = 0.304) or Epoch II (*P* = 0.777). Figure 2 shows hospital survival in neonates with early presentation of respiratory distress for Epoch I and II according to expected survival based on POS score. Within each expected survival category, observed survival was higher during Epoch II.

### Local vs. remote transfer survival

During Epoch II, 44 neonates had early presentation, 31 were local, and 13 were remote transfers. All local transfers, 31 of 31 (100 %), and 11 of 13 (85 %) remote transfers were admitted within 24 h after delivery. Two remote transfers arrived after 24 h from a distant (280 km) hospital via ground ambulance. Of the remote transfers 9 (69 %) survived hospitalization compared to 16 (52 %) of local transfers (*P* = 0.282). Birth characteristics did not differ between local and remote transfers, including gestational age (38.2 ± 2.7 vs. 38.4 ± 3.0 weeks, *P* = 0.900), birth weight (3.1 ± 0.7 vs. 2.9 ± 0.6 kg, *P* = 0.356), Apgar scores at 5 min (6.0 ± 2.2 vs. 4.9 ± 2.7, *P* = 0.193), and POS score (0.60 ± 0.23 vs. 0.46 ± 0.30, *P* = 0.113). However, on admission, local transfers had lower pH (7.01 ± 0.19 vs. 7.26 ± 0.12, *P* <0.001), lower base deficit (−9.8 ± 7.9 mq/L vs. -1.6 ± 4.1 mq/L, *P* = 0.001), and higher PcCO_2_ (87.5 ± 30.1 mmHg vs. 59.8 ± 16.4 mmHg, *P* = 0.003) compared to remote transfers. Also lowest mean blood pressure during the first 12 h after admission was significantly lower in local transfers (34.4 ± 7.2 mmHg vs. 41.2 ± 5.7 mmHg, *P* = 0.005) as was the lowest body temperature (35.8 ± 0.7 °C vs. 36.5 ± 0.5 °C, *P* = 0.002). From a multivariable logistic regression analysis that adjusted for P_c_CO_2_, survival was not found to be significantly associated with the type of transfer (local vs. remote, *P* = 0.997). However, survival significantly improved with lower admission P_c_CO_2_ (OR 1.16, 95 % CI 1.01–1.33 per 5 mmHg decrease, *P* = 0.031).

## Discussion

The main finding of this study is that protective ventilation for newborns with CDH was associated with improvement in hospital survival, primarily due to a substantial increase in survival among high-risk neonates. Despite higher acuity of CDH disease in local neonates compared to remote transfers, the survival was comparable, reflecting high level of care they have received. The level of admission capillary P_c_CO_2_ was an excellent marker for prognostication of survival.

The two most important management changes between epochs were the introduction of protective ventilation and iNO. While iNO is an effective method to control pulmonary hypertension, its use may not reduce CDH mortality [14]. In contrast, ventilator-induced lung injury may substantially increase mortality in neonates with hypoplastic lungs [3, 6–8, 10, 15] while protective ventilation improves survival, and may minimize the need for extracorporeal membrane oxygenation (ECMO) [11]. However, not all studies reported improvement in survival with ECMO [6, 16], and neonatal ECMO was unavailable in Croatia during the study timeframe. In our study the adoption of protective ventilation was associated with a substantial improvement in survival for high-risk neonates with respiratory distress occurring immediately after delivery. Our overall survival in Epoch II was 67 %, which is within the range reported by others [17–21]. However, the true CDH mortality is likely to be higher, as this report does not include “hidden mortality”, i.e. newborns who died before they reached the neonatal unit [22, 23]. A large study reported that 35 % of live-born infants died before being transported to the higher level of care and the population of infants reaching tertiary surgical centers represents approximately 40 % of the total number of cases with CDH [24].

The effect of transport on survival in children with CDH is difficult to assess because of multiple confounders which may introduce patient selection bias. Some studies report higher mortality for outborns (potential bias: more severe cases were transferred and therefore *less* likely to survive) [12, 25], while others describe lower mortality of outborns (potential bias: transferred only less severe cases and therefore more likely to survive) [26, 27]. In the present study, there was no difference in survival between *local and remote transfers*, but these findings also reflect probable referral bias secondary to the availability of resources to transport high acuity neonates and transport distance. We speculate that because of the difficulties in long-distance transfers in Croatia (i.e., use of ground ambulance rather than helicopter) the transfer of CDH newborns from remote locations was reserved for less severe cases, introducing a potential bias towards improved survival. This speculation is supported by our observation that local neonates had more severe derangements in vital signs and had more severe lung hypoplasia as inferred from higher capillary P_c_CO_2_. Arterial P_a_CO_2_ is a good marker for the degree of hypoplastic lung disease. Salas et al. [28] demonstrated that P_a_CO_2_ >88 mmHg on admission (which was the mean P_c_CO_2_ of our local transfers) was associated with low survival, while P_a_CO_2_ <66 mmHg on admission (which was the mean P_c_CO_2_ of our remote transfers) was a marker of improved survival. Our finding of comparable survival between “sicker” local referrals and remote transfers suggests that even the sickest neonates who succeed to reach tertiary care may achieve substantial survival. Since we encountered this imbalance of disease severity between remote and local transfers, our study is limited in drawing definitive conclusions regarding the effect of transport *per se* on survival in neonates with CDH.

### Limitations

The limitation of our retrospective study is possibility for presence of unforeseen confounders. Furthermore, the long time span of the study may hide other unaccounted practice changes that occur over calendar time. In order to examine the effect of calendar time on outcomes additional analyses were done that showed that hospital survival did not increase or decrease over time within either epoch, and the improvement of survival after year 2000 suggests that improved survival can likely be attributed to protective ventilation.

## Conclusions

With the introduction of protective ventilation, survival for high-risk neonates with early respiratory distress substantially improved. Better survival was associated with lower admission capillary P_c_CO_2_. Admission blood gases and vital signs were substantially better in remote transfers indicating on potential referral bias related to transferring neonates with less severe disease. After adjusting for admission P_c_CO_2,_ survival did not differ significantly between local and remote transfers. This suggests that being delivered close to a tertiary care facility may be advantageous, especially for neonates with high disease acuity. Therefore, mothers living in remote rural areas with less specialized neonatal care should be considered for prenatal screening, and if CDH is detected they should be referred for delivery close to an institution with specialized neonatal care.

## Abbreviations

A/C + VL: Assist-control ventilation with volume limit mode
CDH: Congenital diaphragmatic hernia
ECMO: Extracorporeal membrane oxygenation
HFOV: High frequency oscillation ventilation
IMV: Intermittent mandatory ventilation
iNO: Inhaled nitric oxide
NICU: Neonatal intensive care unit
PEEP: Positive end expiratory pressure
PIP: Peak inspiratory pressure
POS: Probability of survival score
PSV + VG: Pressure support ventilation with volume guarantee mode
